# Immune evasion and persistence in enteric bacterial pathogens

**DOI:** 10.1080/19490976.2022.2163839

**Published:** 2023-01-08

**Authors:** Micah J. Worley

**Affiliations:** Department of Biology, University of Louisville, Louisville, Kentucky, USA

**Keywords:** Immune evasion, persistence, *Yersinia*, *Shigella*, *Campylobacter*, *Vibrio*, *Salmonella*, colonization resistance, inflammation

## Abstract

The major function of the mammalian immune system is to prevent and control infections caused by enteropathogens that collectively have altered human destiny. In fact, as the gastrointestinal tissues are the major interface of mammals with the environment, up to 70% of the human immune system is dedicated to patrolling them The defenses are multi-tiered and include the endogenous microflora that mediate colonization resistance as well as physical barriers intended to compartmentalize infections. The gastrointestinal tract and associated lymphoid tissue are also protected by sophisticated interleaved arrays of active innate and adaptive immune defenses. Remarkably, some bacterial enteropathogens have acquired an arsenal of virulence factors with which they neutralize all these formidable barriers to infection, causing disease ranging from mild self-limiting gastroenteritis to in some cases devastating human disease.

## Introduction

The mammalian gut microbiome of healthy individuals is one of the most complex microbial communities ever discovered^1^. In the case of humans, it is composed of trillions of bacteria from over 1000 different species, which are essential to the host in denying pathogens a foothold.^2^ By housing these microbes, the host not only gains nutrients but also a formidable array of defenses against pathogens collectively termed colonization resistance. In addition to housing the endogenous microflora, the gastrointestinal tract and the mucosal tissue provide physical barriers intended to shield the bloodstream and systemic organs from enteropathogens. The dissemination of enteric pathogens to the systemic circulation was traditionally considered passive on the part of microbes but is increasingly being appreciated as an active, microbe-influenced or directed process. The innate immune system discriminates between self and foreign by recognizing certain pathogen-associated molecular patterns (PAMPs) that do not belong, including lipopolysaccharide (LPS), peptidoglycan, flagellin and microbial nucleic acids.^3^ The responsible germline encoded receptors include Toll-like receptors that detect microbial components on the cell surface and Nod-like receptors that serve a similar function within the cytosol.^3^ Following the detection of PAMPs, the pro-inflammatory MAPK and NF-κB signaling pathways are stimulated and inflammasomes are assembled and activated, which are multiprotein signaling complexes that coordinate antimicrobial defenses.^4^ In addition to recognizing PAMPS, the innate immune system can also detect certain conserved pathogen induced processes to distinguish between pathogens and less threatening commensal microbes.^4^ These danger signals activate specific arms of the innate immune system, enabling the host to modulate inflammation according to the threat.^4^ Enteropathogens must strike a careful balance between inducing inflammation in a way that enhances colonization early in infection and suppressing it at later times to prevent premature clearance and to in some cases allow systemic dissemination. There is a shared underlying logic through which enteropathogens surmount the considerable host barriers to infection. All the pathogens discussed here overcome colonization resistance, modulate the proinflammatory MAPK family and NF-κB, both activating them at times and suppressing them or their effects at others to skew the innate immune response to the pathogen’s benefit. They also manipulate cell death pathways in a manner that promotes virulence, triggering either an inflammatory cell death and/or inducing non-inflammatory apoptosis. Finally, they evade adaptive immunity. These aspects of infectious disease along with the emerging theme of virulence genes with allelic variants that dictate how the pathogens harboring them interact with the immune system are discussed. The following subset of prevalent bacterial enteropathogens: *Salmonella, Shigella, Campylobacter, Yersinia* and *Vibrio* are included.

### Salmonella

Of the enteric bacterial pathogens, perhaps the best studied is *Salmonella*. The genus is composed of pathogens of diverse animals as well as humans, responsible for both acute and chronic infections. There are two species: *Salmonella bongori* and *S*. *enterica*. There are several subspecies and over 2,000 serovars. These serovars are typically classified into one of the two groups, those causing typhoid fever in humans and ones that are typically responsible for gastroenteritis. The typhoidal group includes *S*. Typhi, *S*. Paratyphi and *S*. Sendai, all three of which are human restricted. There are over 14 million cases of typhoid and paratyphoid fever per year world-wide.^5^ Some of the more common non-typhoidal serovars that cause disease in humans are *S*. Typhimurium, *S*. Dublin and *S*. Enteritidis. The Centers for Disease Control estimates that there are 1.3 million infections with these serovars in the U.S. alone and invasive disease with them, mostly Typhimurium and Enteritidis, is on the rise and is especially relevant in sub-Saharan Africa, with a high case fatality rate.^6^ This is typically seen in immunocompromised hosts, but also occasionally in the otherwise healthy. This section provides an overview of how *Salmonella* interacts with the immune system in terms of overcoming colonization resistance, manipulating inflammation, overcoming physical barriers, modulating host cell death pathways and what allelic variation contributes to these aspects of its virulence. For a more comprehensive description of all *Salmonella* virulence factors, further reading is suggested.^7–10^

### Animal models of disease

There are a variety of animal models for studying different aspects of *Salmonella* pathogenesis. *S. enterica* infection in these systems produces intestinal inflammation and diarrhea, or acute systemic disease that resembles typhoid fever in humans or chronic systemic disease. The disease symptoms depend on both the serotype of the *Salmonella strain* used and host susceptibility.^11^ Infection of cows with *S*. Typhimurium produces enterocolitis that includes intestinal inflammation and diarrhea.^12^ Both acute and chronic infections of humans with *S*. Typhi can be modeled in mice. *S*. Typhimurium infection of SLC11A1 (NRAMP1) mutant mice with *S*. Typhimurium causes acute systemic disease. Typhimurium literally means Typhi of mice. Infection of wild-type mice with *S*. Typhimurium produces a chronic, systemic disease with long-term carriage as sometimes occurs with *S*. Typhi in humans. Intestinal inflammation and diarrhea are not observed with non-typhoidal *Salmonella* infections of mice unless they are pre-treated with streptomycin.^13^

### Type III secretion systems

*Salmonella* possesses two independent type III secretion systems encoded by *Salmonella* pathogenicity island-1 and *Salmonella* pathogenicity island-2. S. Typhimurium utilizes *Salmonella* pathogenicity island-1 in the gastrointestinal stage of disease to invade enterocytes and invoke acute inflammation that enhances its growth in the lumen of the gut. *Salmonella* was traditionally thought to only deploy *Salmonella* pathogenicity island-2 in the systemic phase of disease, to facilitate intracellular survival and proliferation; however, more recent studies revealed that there is a fair degree of temporal and functional overlap between the two systems.^14–17^

### Overcoming colonization resistance

An inflammatory response is intended to be protective; however, in the case of *S*. Typhimurium infections, it is deliberately induced and enhanced by the pathogen and is crucial for its colonization of the intestinal tract.^18,19^ The inflammation depletes the resident gut microflora of the host enabling *Salmonella* to overcome colonization resistance, by reducing competition for essential nutrients. It also provides *Salmonella* with new carbon sources and electron acceptors that facilitate its outgrowth. It generates reactive oxygen species that produce a new respiratory electron acceptor in tetrathionate that *Salmonella* uses to support its growth on ethanolamine, which cannot be used by competing bacteria.^20,21^ *Salmonella* can use the *Salmonella* pathogenicity island-1 associated effector SopE to generate an energetically more favorable respiratory electron acceptor in host-derived nitrate.^22^ *S. enterica* also overcomes colonization resistance with the deployment of a type VI secretion system encoded with the conserved *Salmonella* pathogenicity island-6. *Salmonella* uses its type 6 secretion system as an antibacterial weapon and it is required for colonization of the mouse gut.^23^ For more information on how *Salmonella* interacts with the gut microbiota, the reader is directed to another recent review.^24^

### Overcoming physical barriers

**Figure 1.**
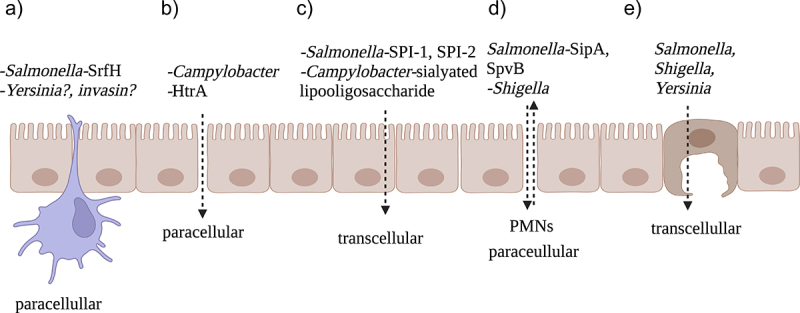
Strategies used by four enteropathogens to breach the gastrointestinal epithelium. A) *Salmonella* and likely *Yersinia* can traverse the physical barrier within CX_3_CR1^+^ phagocytes that send dendrites through the paracellular space without disrupting the tight junctions. SrfH seems to regulate this process and *Yersinia* may require invasin for it to be efficiently utilized. B) *Campylobacter* cleaves E-cadherin, occludin and claudin-8 with HtrA to destabilize tight junctions and pass in between epithelial cells. C) *Salmonella* and *Campylobacter* can also pass through enterocytes. *Salmonella* requires both SPI-1 and SPI-2 effectors for this while *Campylobacter* enhances it with sialylated lipooligosaccharide cell structures. D) *Salmonella* and *Shigella* can recruit polymorphonuclear neutrophils to migrate through the paracellular space to gain access to the submucosa independently of the M cells. E) Finally, *Salmonella, Shigella* and *Yersinia* can pass through the M cells. This figure was generated with Biorender.

Infections with non-typhoidal *Salmonella* are usually self-limiting when confined to the gastrointestinal tract but are fatal 20% of the time with hospitalization after entering the blood.^6^ To reduce fatality, this is the most logical step to therapeutically interdict. *Salmonella* can breach the mucosal barrier, spread to the blood and subsequently internal organs through multiple pathways. The relevant contributions of the various pathways are unclear and in need of additional study. The various strategies used by enteropathogens to breach the gastrointestinal epithelium included in this review are summarized in Figure 1.

*S*. Typhimurium rapidly and preferentially invades the M cells of murine ligated intestinal loops. The destruction of the M cells allows the bacteria to invade adjacent enterocytes.^25^ *Salmonella* infection of the epithelium induces the basolateral secretion of IL-8 that recruits polymorphonuclear neutrophils from the gut associated microvasculature. Tight junctions are integral to the mucosal barrier’s ability to promote intestinal homeostasis, regulating water and solute flow through the paracellular space and preventing the invasion of pathogens.^26^ The type III effector SipA stimulates the synthesis of the chemoattractant hepoxilin A_3_ (HXA_3_), which is secreted apically by the epithelium. The bioactive eicosanoid gradient across the epithelium promotes the transmigration of polymorphonuclear neutrophils from the lamina propria to the luminal side of the barrier through the paracellular space.^27,28^ Interestingly, the ability of SipA to induce HXA_3_ synthesis is confined to the N-terminal domain, while the C-terminal region is responsible for the polymerization of actin, which was shown to be important for invasion into the epithelial cells via ruffling.^29–31^ The two domains are not just functionally distinct but are also physically separated by host caspase-3 cleavage, which may have interesting evolutionary implications.^31^ *Salmonella* further disrupts the tight junctions of the epithelium with the type III effector SpvB.^32^

**Figure 2.**
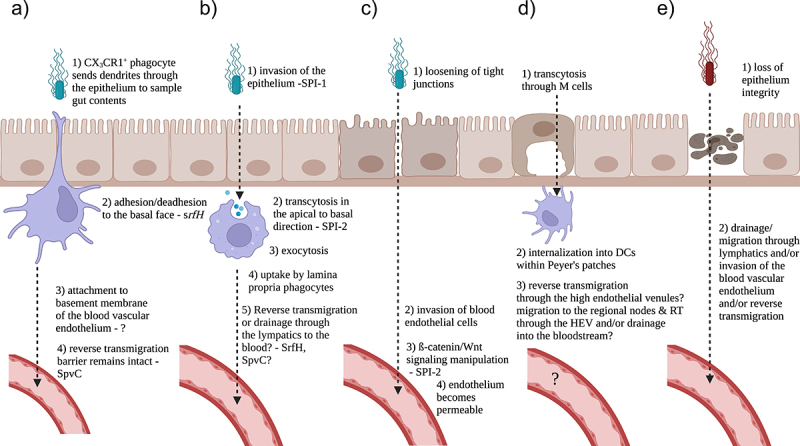
Pathways for *Salmonella* and likely other gut pathogens to spread systemically. A) Reverse transmigration. Infected phagocytes traverse the blood vascular endothelium in the basal to apical direction following uptake of gut bacteria. The tight junctions of the epithelium and the endothelium remain intact. B) *Salmonella* can pass through the epithelial cells and may be able to trigger lamina propria phagocytes to reverse transmigrate or alternatively drain through the lymphatics to the blood. C) *Salmonella* can invade the endothelium and manipulate ß-catenin/Wnt signaling, rendering the blood vessels permeable. D) *Salmonella* can pass through the M cells and drain through the lymphatics to the blood or perhaps trigger the reverse transmigration of infected phagocytes through the high endothelial venules associated with the Peyer’s patches and/or mesenteric lymph nodes. E) *S*. Typhi rapidly destroys epithelial cells and then drains through the lymphatics to the blood and/or triggers the reverse transmigration of infected phagocytes. This illustration was generated with Biorender.

The pathways exploited by *Salmonella* and likely other enteropathogens to spread from the gut to the bloodstream are summarized in Figure 2. Many enteropathogens disseminate to the blood through the lymphatic system. The underlying phagocytes can carry microbes to the regional nodes where secondary immune responses are generated. Microbes that survive within them can eventually drain from the lymph nodes into the systemic circulation inside of them and thereby passively spread throughout the body. Some reports, however, have challenged this classic model for the extraintestinal dissemination of gut pathogens. *Salmonella* and *Yersinia* were found to spread systemically in lymphotoxin ß-receptor knockout mice, which completely lack Peyer’s patches and in congenic control mice similarly.^13,33^ Moreover, some studies have provided evidence that the mesenteric lymph nodes act as a firewall, largely containing oral infections, allowing the generation of a local immune response while shielding the host from systemic, microbial dissemination.^33,34^ While the availability of migratory dendritic cells is the rate-limiting step in mesenteric lymph node colonization, modulation of dendritic cell numbers or migratory properties within the lymphatic system in one such study had no effect on bacteremia.^34^ In this report, the authors showed that while *in vivo* FLT-3 L-induced expansion of dendritic cell numbers, as well as stimulation of dendritic cell migration by TLR agonists, results in increased numbers of *S*. Typhimurium bacteria reaching the mesenteric lymph nodes, there was no increase in microbial dissemination to deeper tissue.^34^ The authors also showed that in CCR7-deficient mice, very few bacteria reach the nodes, but there was no obvious defect in hepatosplenic colonization.^34^ The classic view and this study could in part be explained by the observation of another group of extracellular *Salmonella* autonomously traveling to the mesenteric lymph nodes.^35^ Extracellular *Salmonella* draining through the thoracic duct into the bloodstream would make it unnecessary for intracellular *Salmonella* to modulate the surface expression of host proteins, such as sphingosine-1-phosphate receptor-1. This receptor is up-regulated by the host in response to infection presumably to trap infected cells within the nodes to fight the infection more effectively and guard against sepsis.^36^

Vasquez-Torres *et al*. elegantly defined a pathway of extraintestinal dissemination available to enteropathogens that is independent of the lymphatic system. The authors demonstrated that transgenic mice deficient in CD18, an integrin that mediates leukocyte transmigration, was 100-fold more resistant to the extraintestinal dissemination of a *Salmonella* mutant defective in invading the gut epithelium.^37^ In this alternative pathway, luminal bacteria are internalized by CX3CR1^+^ lamina phagocytes dispersed throughout the lamina propria that send dendrites into the lumen of the gut as a component of antigen sampling.^37^ *Salmonella* and perhaps other enteropathogens can then trigger the infected phagocytes to traverse the blood vascular endothelium in the basal to apical direction without disrupting the tight junctions of the endothelium.^15–17,37,38^ Traversing the blood vascular endothelium in such a fashion without disrupting the tight junctions is termed reverse transmigration when it occurs with uninfected cells, which typically does not occur with infected ones.^15^ With *Salmonella*-infected phagocytes, the process requires the type III effector SpvC.^15^

In a parallel pathway, in the murine model of gastroenteritis, following *Salmonella* pathogenicity island-1 mediated apical invasion of enterocytes, *Salmonella* pathogenicity island-2 type III effectors facilitate the trafficking of the bacteria to the basal side of the epithelium. The bacteria then exocytose to the lamina propria where they are briefly extracellular before being internalized by phagocytes.^39^ The authors proposed that phagocytes sample the contents of the gastrointestinal epithelium as part of a constitutive antigen sampling pathway that *Salmonella* exploits.^39^ It would be interesting and important to assess in a different model of disease if *Salmonella* colonized the lamina propria and perhaps accessed deeper tissue. This could occur by infected phagocytes draining through the portal vein or the thoracic duct or reverse transmigrating through the gut associated blood microvasculature.

In another pathway, in the murine model of typhoid fever, *Salmonella* can access the blood after increasing the permeability of the blood vasculature associated with the gut by manipulating ß-catenin/Wnt signaling with *Salmonella* pathogenicity island-2.^40^ When transgenic mice expressing a degradation resistant ß-catenin allele within endothelial cells were infected with *Salmonella*, no extracellular dissemination was observed. ß-catenin/Wnt signaling, however, affects many diverse cellular processes.^41^

There are clearly myriad ways in which enteropathogens can disseminate extra-intestinally and an important challenge for the field is assessing the relative contributions of each. A limitation in many of these studies is that when one pathway is blocked and no defect is observed, more of the inoculum may simply go through the other pathways if they are not normally saturated. This may mask the contribution of the one that is unavailable under certain conditions. The interpretation of these studies is further complicated by the use of mouse and bacterial strains of different genetic backgrounds as well as the use of different time points.

It is interesting to consider that there may be pathways to the blood from the gut available to enteropathogens that remain to be discovered. When CD18 mutant mice are infected with *Salmonella* pathogenicity island-1 mutant bacteria that are deficient in invasion, *Salmonella* is still able to colonize hepatosplenic tissue in significant numbers.^37^ This suggests the existence of an additional pathway to the bloodstream that does not require reverse transmigration or *Salmonella* pathogenicity island-1 mediated invasion of the epithelium or endothelium. Two possibilities are the invasins PagN and Rck, which can facilitate invasion of epithelial cells independently of *Salmonella* pathogenicity island-1.^42,43^ A unifying observation is that *Salmonella* pathogenicity island-2 and the anti-inflammatory effectors SpvC and SpvD are required for any systemic dissemination in the murine model of *Salmonella* infection, even though they are dispensable for intracellular survival.^15,44–46^ They also appear to be required to cause bacteremia in humans with non-typhoidal serovars.^15,44,47–49^

### Activation and suppression of inflammation

The pathogen must strike a careful balance between inducing inflammation initially and later dampening it to prevent the infection from being prematurely cleared and to allow dissemination to deeper tissue. Non-typhoidal serovars typically trigger inflammatory responses, whereas the typhoidal ones suppress it. *Salmonella*, like other enteric bacterial pathogens, possesses a large repertoire of secreted effectors, which either induce or down regulate the inflammatory response of infected hosts. *S*. Typhimurium must bypass the negative regulatory mechanisms of the host intended to prevent constitutive activation of innate immune receptors in the gut. The mechanisms presumably exist to prevent chronic inflammation resulting from exposure to common bacterial products from harmless members of the microflora. This is presumably to minimize the chances of autoimmune disorders such as Crohn’s disease or inflammatory bowel disease.

The features of the more prominent pro-inflammatory effectors of the five genera of enteric bacterial pathogens included in this review are summarized in Table 1. The three *Salmonella* pathogenicity island-1 effectors SopE, SopE2 and SopB circumvent host inflammation attenuating mechanisms by activating the Rho family GTPases RAC1 and CDC4 and a non-canonical signaling complex downstream of Toll-like receptors.^50,53,54,76,77^ This ultimately leads to MAPK and NF-κB signaling and the production of pro-inflammatory cytokines. Importantly, inhibiting the downstream signaling hub attenuates the growth of *S*. Typhimurium in the gut but increases the number of bacteria that translocate to systemic sites.^53^

**Table 1.** Major pro-inflammatory effectors.

Organism	Effector	Function	Effect on host	References
*Salmonella*				
	SopB	Phosphoinositide phosphatase	Activates for Rho-family GTPase GEFs	^50,51^
	SopE/SopE2	GEFs for Rho Family GTPases	Induce NF-κB signaling	^52–54^
	SopA	E3 ubiquitin ligase	Ubiquitinates TRIM56 and TRIM65 to activate RIG-I and MDA5 signaling	^55^
	SopD	RAB8 GAP	Supresses RAB8-dependent anti-inflammatory pathway	^56^
*Shigella*				
	IpgB1	Rac1 GEF	Activates NF-κB signaling	^57^
	IpgB2	Rac1 GEF		
	OspB	Activates p38, ERK1/2, and PLA2 early in infection	Stimulates the secretion of chemoattractant and IL-8	^58,59^
	OspZ_1-188_	Phosphorylates ERK		^60^
	OspC1	Phosphorylates ERK1/2	Promotes PMN recruitment to the epithelium	^61^
*Campylobacter*				
	CiaD	Activates MAPKs	Secretion of IL-8 among other things	^62^
	CDT	PIP3 phosphatase	PI-3 K blockade results in pro-inflammatory signaling	^63,64^
	guide-free Cas9	Reprograms epithelial cells by activating DNA damage	Promotes NF-κB signaling	^65,66^
*Yersinia*				
	YopE	GAP for Rho GTPases Rac1, RhoA & Cdc42	Blocks internalization and activates pyrin	^67–69^
	YopT	Cysteine protease that targets RhoA	Blocks internalization and activates pyrin	^67–72^
*Vibrio*				
	HlyA (cytolysin)	Pore forming toxin	Activates NF-κB and MAPK signaling, induced apopostis	^73,74^
	Outer membrane vesicle cargo		Induces inflammation	^75^

The effectors SopA and SopD amplify the inflammatory responses induced by SopE, SopE2 and SopB. SopA is a ubiquitin ligase that activates TRIM56 and TRIP65 to induce interferon genes and inflammation.^78^ SopD enhances inflammation without engaging innate immune receptors. This effector interdicts an anti-inflammatory pathway downstream of Toll-like receptors that the host uses to limit tissue damage after fighting an infection that depends on RAB8 and AKT.^79,80^

*Salmonella* possesses mechanisms to not only induce inflammation to promote colonization and replication but also the ability to attenuate it. The functions of the more prominent effectors of the five enteropathogens discussed in this review that reign in the inflammatory response elicited by other effectors are described in Table 2. Preserving host homeostasis may seem counterintuitive at first. They may have evolved to promote a long-standing association with the host. They also may dampen inflammation in a spatiotemporally regulated fashion to allow intracellular pathogens capable of causing systemic illness the opportunity to spread to deeper tissue after successfully overcoming colonization resistance. Deleting some type III effectors results in diminished intestinal disease but enhances spread to systemic sites^84,111,112^ These effectors antagonize the actions of the pro-inflammatory ones by directly countering their effects or alternatively by activating anti-inflammatory pathways.

**Table 2.** Major anti-inflammatory effectors.

Organism	Effector	Function	Effect on host	References
*Salmonella*	SptP	Rho family GTPase GAP	Opposes the effects of SopE/SopE2	^81^
	SopB	Phosphoinositide phosphatase	Stimulates PI3K -dependent anti-inflammatory signaling	^51,56^
	SopD	GDI-dissociation factor for RAB8	Stimulates PI3K -dependent anti-inflammatory signaling	^56^
	SteE/SarA	Forms a complex with STAT3	Stimulates STAT3-dependent anti-inflammatory signaling	^82,83^
	PipA, GtgA, GogA	Proteases for NF-κB transcription factors	Inhibit NF-κB-dependent transcription	^84^
	AvrA	Acetylates MKK4 and MKK7	Inhibits JNK signaling	^85,86^
	SseK1, SseK3	N-Acetylglucosamine transferase for DEAD-domain containing proteins	Inhibits NF-κB signaling	^87,88^
	SpvB	Down-regulates IKKβ	Inhibits NF-κB signaling	^89^
	SpvC	Phosphothreonine lyase for ERK1, ERK2 and p38	Prevents MAPK signaling	^47,90^
	SpvD	Inhibits RELA nuclear translocation	Inhibits NF-κB signaling	^91^
	SrfH/SseI	Directly or indirectly dephosphorylates Erk2	Some alleles promote infected host cell deadhesion/motility	^16,17,92^
	GogB	inhibitis IκBα degradation	Inhibits NF-κB signaling	^93^
*Shigella*				
	OspF	Phosphothreonine lyase	Inactivates MAPKs	^90^
	IpaH7.8	Stimulates the degradation of human, but not murine gasdermin D	Blocks pyroptosis of human cells	^94^
	IpaH1.4 & IpaH2.5	E3 ligases	attenuate NF-κB nuclear translocation	^95^
	IpaH9.8	Targets NEMO/IKKγ & a subset of guanylate-binding proteins & a splicing factor	Effects all targets in manner that dampens inflammation	^96–98^
	IpaH4.5	Targets TBK1 and thus the IFN regulatory factors	Attenuates inflammation and antibacterial response	^99^
	IpaH0722	Targets TRAF2	Inhibits NF-κB activity	^100^
	OspG	Ser, Thr kinase that prevents the ubiquitination of phospho-IκBα	Inhibits NF-κB activity	^101,102^
	OspI	Deamidates UBC13	blocks TRAF6-dependent NF-κB signaling	^103^
	OspZ	Blocks The nuclear localization of the NF-κB subunit p65	Prevents NF-κB activity	^60^
*Campylobacter*				
	Not yet identified	Activates PI-3 K/Akt1 pathway	Induces an anti-inflammatory response	^104^
*Yersinia*	YopM	Subverts negative regulators of pyrin to counter the pro-inflammatory activities of YopE and YopT	Down-regulates pro-inflammatory cytokines	^105–107^
	YopK/YopQ	prevents hyper translocation of the T3SS pore-forming proteins	Decreases inflammasome activation	^108,109^
*V. cholerae*	DNAases	Degrade NETs	Combats inflammation by reducing the chance of entrapment	^110^
	Outer membrane vesicle cargo	Increase levels of microRNA miR-146a	Attenuates the inflammation induced by cytolosin	^75^

SptP is a member of the first group, which serves as a GTPase activating protein for CDC42, RAC1 and RhoA. It directly counters the effectors SopE and SopE2 that are guanine nucleotides exchange factors for these GTPases.^81^ A subset of effectors composed of PipA, GtgA and GogA form a family of related proteases that cleave RELA and RELB, which are transcription factors for NF-κB.^84^ The importance of limiting the inflammatory response in this fashion is indicated by the presence of at least one member of this set of genes in all characterized *Salmonella* isolates.^84^

The effectors SseK1 and SseK3 also inhibit NF-κB signaling albeit with a different mechanism. These two effectors are arginine glycosyltransferase that modifies the death domains of proteins in the NF-κB signaling pathway.^87,88^ The *S*. Typhimurium effector protein AvrA possesses acetyltransferase activity toward MAPKKs that attenuate inflammation by inhibiting JNK and NF-κB signaling.^85,86^

Some *Salmonella* serovars carry plasmids, which share a highly conserved locus called the *spv* (*Salmonella* plasmid virulence) operon. In addition to acting on tight junctions, SpvB depolymerizes actin and was recently reported to inhibit NF-κB activity by downregulating IKKβ.^89^ SpvC is a phosphothreonine lyase that dephosphorylates the MAP kinases Erk1/2, p38 and JNK.^90^ SpvC is not required for survival within macrophages or the gastrointestinal tract but is necessary for non-typhoidal *Salmonella* to cause bacteremia in mice and humans.^15,44,49^ SpvD is a cysteine hydrolase with a serovar-specific polymorphism that determines the degree to which it inhibits nuclear transport of NF-κB p65.^91,113^

An often-overlooked component of an inflammatory response is the inhibition of the movement of infected cells. *Salmonella* neutralizes this host checkpoint intended to prevent the spread of pathogen within a host in part with SpvC. This allows it to reach privileged sites of infection with reverse transmigration and likely with enhanced migration through the lymphatic system as well. It will be important to test if SpvC stimulates the migration of infected phagocytes through lymphatic vessels toward the nodes and/or reverse transmigration through the high endothelial venules associated with the Peyer’s patches and mesenteric lymph nodes.

A recent report established SopD as the newest member of an emerging class of bifunctional virulence factors that remarkably possess seemingly opposed activities, which raises interesting questions about the regulation of their activities and/or targets. SopD both induces and limits inflammation. It inhibits Rab8 with GTPase activity. This stimulates inflammation, but it can also activate it by displacing Rab8 from its cognate guanosine dissociation inhibitor, which is anti-inflammatory.^56^ Both SopD and SopB activate a RAB8-dependent PI3K–PKB–mTOR pathway that is downstream of Toll-like receptors, which results in the production of the anti-inflammatory effector, IL-10.^56,114–116^

The effector SteE/SarA/GogC was recently shown in a pair of reports to mimic a cytokine receptor’s intracellular domain and reprogram a non-canonical downstream effector to induce the expression of anti-inflammatory rather than pro-inflammatory genes.^82,83^ SteE specifically targets signal transducer and activator of transcription 3 (STAT3), which the host uses among other things to recover after launching an inflammatory response.^117,118^ Interestingly, the mechanism does not involve the Janus kinases but rather the host kinase GSK3 that phosphorylates SteE.^82,83^

### Modulation of host cell death

*Salmonella* modulates three different host cell death pathways, either inducing them or suppressing them, including apoptotic, necroptotic and pyroptotic ones to promote persistence.^119^ Ultimately, *Salmonella*-infected epithelial cells undergo pyroptosis, which releases proinflammatory cytokines and releases the bacteria. It actively invades macrophages next to reduce the likelihood that it is internalized and killed by more microbicidal neutrophils. Several *Salmonella* pathogenicity island-1 and *Salmonella* pathogenicity island-2 secreted type III effectors have been implicated in inducing either rapid or delayed apoptosis of macrophages.^120–122^ The two events were speculated to be either temporally or anatomically regulated within hosts and are genetically separable.^121^ After inducing cell death, apoptotic cells harboring *Salmonella* are engulfed by new macrophages, creating a fresh intracellular niche for the pathogen, allowing continued persistence and avoidance of extracellular host defenses. For more information on the manipulation of host cell death pathways by *Salmonella*, another review is suggested.^119^

### Evasion of adaptive immune responses

In addition to subverting innate immunity, *Salmonella* also delays an adaptive immune response. *S*. Typhimurium can kill CD8α^+^ dendritic cells in mesenteric lymph nodes and can inhibit T cell proliferation by a direct, contact-dependent immunosuppressive effect, which inhibits the ability of T cells to produce cytokines and proliferate.^123,124^ CD4^+^ T cells specific for *Salmonella* have been identified shortly following infection; however, they do not play a role in battling the infection until several weeks later and this delay requires *Salmonella* pathogenicity island-2. Several *Salmonella* pathogenicity island-2 effectors interfere with antigen presentation by dendritic cells and in the case of SteD ubiquitinates MHC-II molecules.^8,125,126^ In addition to downregulating antigen presentation by dendritic cells, *Salmonella* pathogenicity island-2 actively induces the apoptosis of antigen-specific CD4^+^ T cells.^127^ For additional information on the evasion of adaptive immunity by *Salmonella*, additional reading is suggested.^8,125^

### Persistent infections

One of the most interesting disease manifestations of host adapted strains of *Salmonella* in mammals is that of an asymptomatic, chronic infection. The bacteria overcome the formidable defenses of the gastrointestinal tract and lymphatic system to ultimately reside within macrophages at systemic sites for in some cases the lifetime of the host. This is a highly advantageous niche for the bacteria that are generally free of endogenous flora and rich in nutrients, and moreover the pathogen can be intermittently shed. Biofilm formation on the surface of gallstones is associated with the establishment and perpetuation of such infections with *S*. Typhi.^128^ These infections are largely asymptomatic but can be a contributing factor in the development of gallbladder cancer.^128^ The carriers are also a public health concern as the pools of bacteria may allow the generation of new genotypes, and they serve as reservoirs for dissemination to new hosts.^129^

Once within macrophages at systemic sites, some *S*. Typhimurium cells enter a non-growing but transcriptionally and translationally active state that allows for long-term, chronic infections that are resistant to antibiotic treatment. These cells trigger a non-inflammatory M2 polarization of the infected macrophages with the *Salmonella* pathogenicity island-2 effector SteE, which antagonizes TNF-mediated pathogen restriction.^130,131^ In addition to manipulating the immune status of these cells, *Salmonella* also metabolically reprograms them to create a favorable environment for bacterial persistence. Among other changes, these cells are induced to express a high level of PPARS, a eukaryotic transcription factor involved in fatty acid metabolism, by increasing the availability of glucose.^132^ For additional information on persistent *Salmonella* infections, other reviews are recommended.^129,133,134^

A growing problem with *Salmonella* is the multi-drug resistance persistence strategy that is employed by all enteropathogens. The public health relevance of the typhoidal serovars is increasing with the rise of such resistance, seen in >60% of strains.^135^ In addition to the persister state described above, strains of typhoidal *Salmonella* that have acquired specific resistance mechanisms to chloramphenicol, ampicillin, and trimethoprim have caused numerous outbreaks.^135^ As a result of the widespread dissemination of such strains, chloramphenicol was withdrawn as the first-line drug for typhoid fever and replaced with fluoroquinolones and third generation cephalosporins.^136^ However, outbreaks of typhoid fever caused by strains resistant to nalidixic acid and ciprofloxacin have become endemic in the Indian subcontinent and have also been reported in the US and UK among other developed countries, reflecting the emergence of a global problem.^137^ The presence of a plasmid-borne integron in ciprofloxacin-resistant *S. typhi* may soon produce widespread instances of nearly intractable typhoid fever.^137^

In a genome-wide screen for *S*. Typhimurium genes that promote chronic disease in a mouse model of long-term systemic infections, dozens of genes were implicated in facilitating chronic infections.^138^ These genes are contained within *Salmonella* pathogenicity island-1, 2, 3, 4, 5 and 6.^138^ Fimbriae and genes within the integrated phages GIFSY-1 and GIFSY-2 among others were also identified.^138^ Many of the genes are of unknown or putative function. A *Salmonella* pathogenicity island-2 associated effector identified of particular interest in causing persistent infections is SrfH/SseI.^138^ Some alleles of SrfH can deamidate the heterotrimeric G protein Gαi2, resulting in persistent activation through non-polarized activation of it.^139^ This results in the loss of directed dendritic cell migration, perhaps through increased adhesion.^139,140^ This promotes the long-term colonization of wild-type mice, which do not typically succumb to infection as C57Bl/6 mice do.^140^ This is perhaps due to a reduction in the directed migration of phagocytes along T cell chemoattractive gradients in systemic organs.^140^ It also may suppress the de-adhesion and migration of infected cells within the lymphatic system and of antigen sampling dendritic cells in the reverse transmigration pathway.^16,17,140,141^

### Role of allelic variants in infection dynamics

A fair amount of attention has been devoted to understanding what differences in gene content allow enteric microbes to cause disease versus non-pathogenic relatives. Additionally, studies have identified and characterized what such differences contribute to microbes from the same genera causing different types of disease, often differing in severity. However, very little has been invested in identifying and characterizing how changes between various alleles of the same genes influence virulence. Understanding how differences among naturally occurring alleles of virulence genes affect how the pathogens harboring them interact with the immune system and persist within hosts is an area in need of more study.

SrfH and SpvD were the first two of over a dozen genes for which non-synonymous differences among naturally occurring alleles were determined to influence whether *Salmonella* escapes confinement within the gastrointestinal tract.^16,142^ In a bioinformatics study with non-typhoidal *Salmonella*, either a dominant or a recessive allele of 22 genes was associated with invasive versus gastrointestinal disease.^142^ Most of these genes play roles in mediating adhesion or inflammation. From the 22 effectors for which known allelic variants are associated with a particular phase of disease, a subset of three: SspH2, SlrP and SrfH were shown to inhibit dendritic cell chemotaxis toward CCL19 in a microfluidic device.^143^ It is interesting to consider that anti-inflammatory effects have been attributed to all three effectors in some reports.^92,144,145^

SrfH is particularly interesting because of the potential of some alleles, as with SopD, to possess not just bifunctional, but seemingly opposed activities. In strains primarily associated with gastrointestinal disease in humans, the catalytic triad in the C-terminus appears to suppress deadhesion, which can be at least partially alleviated with certain polymorphisms in the N-terminus found in more invasive strains and/or with ones within the catalytic site of the carboxyl terminus.^16,17,139–142^ Intriguingly, hyper-invasive sequence type 313 isolates, which are responsible for epidemic multiple drug resistant invasive disease with *S*. Typhimurium in Sub-Sahara Africa, possess a disrupted SrfH allele.^141^ This pseudogene contains the catalytic triad of the carboxyl terminus necessary for Gαi2 deamidation but lacks any of the compensatory polymorphisms seen in other highly invasive strains that seem to mask the effect of the carboxyl region on adhesion.^140–142^ The different effects on adhesion and motility and extraintestinal dissemination reported for SrfH in various reports are likely attributable to the use of strains possessing different alleles of this intriguing effector.^16,17,140^

### Shigella

*Shigella* is responsible for an estimated 125 million diarrheal episodes annually that result in 160,000 deaths and is especially prevalent among young children.^146^ The resulting disease, termed Shigellosis, is characterized by watery diarrhea, abdominal cramps and fever. The *Shigella* genus is composed of four major species: *Shigella dysenteriae, Shigella flexneri, Shigella boydii*, and *Shigella sonnei. S. dysenteriae* and *S. flexneri* are associated with poverty and poor hygiene, while *S. sonnei* is more common in affluent regions. Considering the low infective dose, on the order of 10 to 100 organisms, associated disease severity and increasing antimicrobial resistance, preventing shigellosis is a major public health issue. *Shigella* promotes its pathogenesis with chromosomal pathogenicity islands and a large virulence plasmid that harbors a type III secretion system (Mxi-Spa) and numerous effectors, including several invasion plasmid antigens (Ipas). These genomic features are key to its manipulation and neutralization of innate and adaptive host defenses. This section provides an overview of how *Shigella* interacts with the immune system in terms of overcoming colonization resistance, manipulating inflammation, evading adaptive immunity and modulating host cell death pathways. For a comprehensive discussion of all *Shigella* virulence factors, the reader is referred to other reviews.^96,147–149^

### Animal models of shigellosis

Bacillary dysentery is a human-specific disease that follows the triggering of a strong inflammatory response after *Shigella* invades the colonic epithelium. A drawback in *Shigella* pathogenesis studies has been the lack of an appropriate animal model that mimics the natural course of infection and immune response in humans. Adult mice are refractory to oral infection; however, murine models were developed with intraperitoneal, as well as oral infection that involves a zinc deficient diet and pre-treatment with antibiotics.^150,151^ There is also an infant rabbit model available as is a guinea pig model that requires the intrarectal administration of bacteria.^152,153^

### Overcoming colonization resistance

*Shigella* is inadvertently ingested through the fecal-oral route with contaminated food or water. The low infectious dose of *Shigella* indicates that it possesses potent mechanisms for overcoming colonization resistance. *Shigell*a withstands gastric acid better than some of the other enteric pathogens, due primarily to its glutamate decarboxylase system.^154^ *Shigella* disrupts the production of bactericidal peptides at the surface of the gastrointestinal epithelium, which is an important component of innate immunity.^155^ *Shigella* resists colicins with WzzB, an O-antigen chain length regulator and moreover secretes colicins of its own.^156^ *Shigella* enterotoxin 1 (ShET1) and *Shigella* enterotoxin 2 (ShET2) induce fluid secretion in the jejunum to establish infection. It produces the watery diarrhea seen early in shigellosis. There is no homology between ShET1 and ShET2. ShET1 is encoded by *set1A* and *set1B* genes contained within a chromosomal pathogenicity island only present in *S. flexneri* 2a isolates and are believed to form a holo-AB-type toxin complex.^157,158^ ShET2 is a 63 kDa protein encoded by *ospD3* that induces IL-8 secretion by epithelial cells and is found in all serotypes.^159^ ShET2 but not ShET1 is secreted by the type III secretion system.^110^ *S. sonnei* can additionally kill members of the endogenous microflora with a type VI secretion system and because of it outcompetes *S. flexneri*, which lacks it, in mixed infections of mice.^160^ This secretion system may explain the increasing prevalence of dysentery caused by *S. sonnei* at the expense of S. *flexneri*, seen with global development.

### Overcoming physical barriers

*Shigella* targets the M cells for transit through the epithelium. Following its release from the underlying macrophages, the bacteria can induce its internalization into enterocytes by triggering the reorganization of the cytoskeleton resulting in a macropinocytic event and subsequently spreads intra- and intercellularly. Recently, a bimodal model was proposed for the invasion of the colonic epithelium, following studies on a human *in vitro* M cell model. In this system, *Shigella* either rapidly transited the M cells or alternatively, perhaps to evade some of the immune surveillance functions of these cells, spreads laterally from the M cells to neighboring enterocytes.^161^

Upon internalization within host cells, *Shigella* lyses the phagosome it is initially contained within to reach the cytosol, its preferred replicative niche, sheltered from many components of the immune system. *Shigella* initially re-programs enterocyte gene expression toward a strong pro-inflammatory profile that promotes polymorphonuclear neutrophil infiltration.^27^ The integrity of the epithelium is destroyed by the polymorphonuclear neutrophils recruited by *Shigella* infection, which allow more luminal bacteria access to the submucosa independently of the M cells. This massive inflammation and destruction of the epithelium is necessary for the development of watery diarrhea and greatly benefits *Shigella* initially but the polymorphonuclear neutrophils ultimately resolve the infection and thus *Shigella* down-regulates inflammation in the final stage of infection.

### Modulation of host cell death

*Shigella* can quickly kill submucosal macrophages by triggering pyroptosis, which frees the bacteria to invade the basal face of the colonic epithelium, but also invokes an intense inflammatory response.^162^ Intriguingly, *Shigella* can also trigger a non-inflammatory macrophage apoptosis with IpaD.^163^ *Shigella* secretes IpgD, which is homologous to Salmonella SopB into infected epithelial cell cytosol, where it dephosphorylates phosphatidylinositol 4,5-bisphosphate (PIP2) into phosphatidylinositol 5-phosphate (PI5P).^164^ IpgD promotes host cell survival because PIP5 among other things activates AKT1.^165^ The existence of both parallel pathways may be attributable to *Shigella* striking a delicate balance between infectiousness and immune evasion.

There are three forms of cell death regulated by intestinal epithelial cells that are important components of the innate immune response to intracellular bacteria. There is crosstalk among the three pathways to deny pathogens an intracellular niche even if they can suppress one of them. Various inflammasome complexes can initiate a lytic inflammatory cell death termed pyroptosis that results from caspase-1/caspase-4-mediated generation of gasdermin pores, whereas caspase-8/caspase-9 activation induces apoptosis. A lytic necroptotic death follows phosphorylation and trimerization of MLKL that causes membrane destabilization. In murine epithelial cells, loss of caspase-8, which prevents apoptosis, will lead to necroptotic cell death.^166^

Regardless of the host cell death pathway redundancies, *Shigella* can prevent all forms of regulated epithelial cell death. *Shigella* uses the T3SS effector OspC3 early in infection to prevent caspase-4-dependent pyroptotic death.^167^ Later, OspC1 interferes with the induction of apoptosis by preventing caspase-8 activation. The host senses caspase-8 inhibition and as a fail-safe induces necroptosis as a backup cell death pathway. It attempts to do this with RIPK/RIPK3 interaction and subsequent phosphorylation of MLKL, but *Shigella* deploys OspD3 to disrupt this pathway by cleaving RIPK1 and RIPK3.^168^ Collectively, the actions of these effectors prevent the host from denying *Shigella* an intracellular niche. For more information on the modulation of host cell death by *Shigella*, a recent review is suggested.^169^

### Overcoming innate defenses

Once free in the epithelial cytosol, *Shigella* inhibits ER to Golgi traffic with VirA and IpaJ, which mainly inactivate Rab1 and ARF6, respectively, to escape autophagic host defense.^170,171^ Spreading intercellularly within the colonic epithelium is important for renewing *Shigella*’s preferred replicative niche and for evading immunological detection. IcsA (VirG) is important for actin-based motility and spread within the epithelium. Actin-based motility mediated by this autotransporter results in the membrane protrusions that allow the invasion of one cell in an epithelium from an adjacent one.^172^ IcsB prevents sequestration by autophagy that would be triggered by the effector IcsA by camouflaging its Atg5 binding site.^173^

The bacteria neutralize the innate defense against invading microbes of rapid mucosal epithelium turnover and exfoliation by hijacking integrin-linked kinase with the virulence factor OspE3 to stabilize focal adhesions and block cell detachment.^174^ OspE cognate genes are conserved among many enteropathogens, including enteropathogenic *E. coli*, enterohaemorrhagic *E. coli, Citrobacter rodentium* and *Salmonella*.^149^ *Shigella* deploys IpaB to further preserve its cytosolic niche by inhibiting cell division. *Shigella* initially re-programs enterocyte gene expression toward a strong pro-inflammatory profile that promotes polymorphonuclear neutrophil infiltration.^175^

### Activation and suppression of inflammation

*Shigella* skillfully skews the immune response to infection in opposing ways in a temporal fashion. Initially, some type III effectors are secreted to phosphorylate and activate MAPK pathways.^149^ The ensuant increased apical secretion of the chemoattractant IL-8 results in polymorphonuclear neutrophil transmigration across the epithelium, destabilizing the barrier and allowing luminal *Shigella* access to the submucosa independently of the M cells. The massive inflammation promotes early infection, but the bacteria subsequently secrete effectors that downregulate proinflammatory signals, perhaps to balance the severity of the inflammation to a level beneficial for the bacteria and to render the host partially susceptible to re-infection. IpgB1 and IpgB2 are guanine nucleotide exchange factors (GEF) that target Rac1 and RhoA, respectively, which results in membrane ruffling.^57^ Interestingly, IpgB2 can activate NOD1 signaling with ensuant NF-κB activation independently of its GEF activity.^176^

In the instance of OspF, similar to *Salmonella* SopB, it may both increase and dampen inflammation with the same effector at different times during the infection. The *S. flexneri* type III effector protein OspF is homologous to *Salmonella* SpvC and Pseudomonas syringae HopAI.^177^ This family of enzymes permanently deactivates the MAPK signaling pathways with phosphothreonine lyase activity.^90^ Additionally, in the guinea pig model of Shigellosis, OspF diminishes the phosphorylation of HP1γ, a member of the heterochromatin protein 1 family of epigenetic regulators that modulate the amplitude of the innate immune response.^178^ OspF, in addition to attenuating inflammation, interestingly has also been attributed pro-inflammatory roles.^179^ This is yet another example of the same effector having opposite effects on the same host process at different times. It will be important to identify all the targets of OspF and to determine how these targets and/or the activities of this effector are regulated.

### OspB

OspB is a type III effector that stimulates inflammation by activating p38, ERK1/2, and phospholipase A2 (PLA2). PLA2 stimulates the secretion of chemoattractant and IL-8.^58^ PLA2 activation also mediates eicosanoid generation, which enhances inflammation.^180^ OspB also modifies the intracellular niche to the advantage of *Shigella* by manipulating the mechanistic target of rapamycin complex 1, which is a master regulator of cell growth and proliferation.^181^

### IpaH family

The IpaH family of type III effectors is composed of 12 members that reside on either the chromosome or the large plasmid that target NF-κB signaling.^182^ These virulence factors possess N-terminal leucine-rich repeats (LRR) that are recognized as a pathogen-associated molecular pattern by the infected host cell and conserved E3 ubiquitin ligase activity in their C-terminal regions.^182,183^ While the members of this family of *Shigella* effectors share homology, their LRR regions, which are the substrate recognition sites, differ as does their subcellular localization. This implies that they have different host targets and make distinct contributions to disease. A variety of pathogens have homologs of the IpaH family, including *Edwardsiella, Bradyrhizobium, Rhizobium*, and some *Pseudomonas* species and also *Salmonella* (SspH1, SspH2, SspH3 and SlrP).^182,184^

*Shigella* IpaH7.8 is an interesting member of the IpaH family of ubiquitin ligases, which stimulates the proteasomal degradation of human but not murine gasdermin D, thereby blocking pyroptosis in *Shigella*-infected human cells. It appears to function differently in mouse cells.^94^IpaH1.4 and IpaH2.5 are homologous E3 ligases that irreversibly inactivate the catalytic subunit of the linear ubiquitin chain assembly complex, HOIP. Normally, the association of the linear ubiquitin chain assembly complex with activated cytokine or pattern recognition receptors results in the ligation of ubiquitin chains to RIPK1 and IKKγ. This event is necessary for the activation of NF-κB. Thus, these two effectors are anti-inflammatory because they attenuate NF-κB nuclear translocation in response to pathogen-associated molecular patterns, TNF as well as IL-1β.^95^

IpaH9.8 dampens NF-κB signaling by targeting NEMO/IKKγ and also ubiquitinates a subset of guanylate-binding proteins which are members of the dynamin superfamily of GTPases important for protecting cells from intracellular pathogens that escape into the cytosol.^96–98^ IpaH9.8 was also reported to bind to a splicing factor whose depletion from cells lowers the levels of pro-inflammatory cytokines.^185^

Among other targets for the ubiquitin ligase IpaH4.5 is TANK-binding kinase 1 (TBK1). TBK1 is involved in the regulation of IFN regulatory factors and activates interferon regulatory factor 3 in response to infection. IpaH4.5 promotes the K48-linked polyubiquitylation of TBK1 resulting in its proteasomal degradation and thereby attenuates the host antibacterial response.^99^ IpaH0722 inhibits NF-κB activity by targeting TRAF2 for degradation, which lies between PKC and NF-κB.^100^

The OspC family, OspI, OspZ and OspG, also has anti-inflammatory effects. The OspC type III effectors were recently shown to block interferon signaling independently of inhibiting cell death by blocking calmodulin kinase II and downstream JAK/STAT signaling and preventing STAT1 phosphorylation.^186^ OspG is a serine/threonine kinase homologous to EPEC NleH that prevents the ubiquitination of phospho-IκBα that is necessary for NF-κB activity.^101,102^ OspI deamidates UBC13 to blocks TRAF6-dependent NF-κB signaling.^103^ The nuclear localization of the NF-κB subunit p65 is blocked by OspZ.^60^

### Evading adaptive immune responses

*Shigella* targets lymphocytes, including activated human peripheral blood B cells, CD4^+^ T, and CD8^+^ T lymphocytes as well as switched memory B cells with type III effectors without triggering internalization.^187^ This direct targeting of lymphocytes independent of invasion may contribute to an inefficient priming of the adaptive immune response. *Shigella* infection of human B and T lymphocytes impairs T cell chemotaxis and the ability of these cells to scan antigen presenting cells. This may be attributable to the targeting of intracellular vesicular trafficking by IpaJ and VirA.^188^

B cells were shown to undergo apoptosis independently of both invasion and type III effector injection following the activation of TLR2-dependent signaling by the type III secretion system needle-tip protein, which was termed the ‘kiss-and-run’ strategy.^189,190^ This may delay the adaptive immune response of mucosal sIgAs and systemic IgGs, which can mediate immunity to *Shigella*. Immunity to *Shigella* infection is ultimately due to an immune response against the LPS O-antigen and Ipa proteins among other molecules.^190^ *Shigella* evades host immunity and renders a recovered host at least partially susceptible to reinfection by varying the composition of the O-antigens across strains and serotypes.^190^ In addition to TBK-1, the ubiquitin ligase IpaH 4.5 also induces the degradation of the proteasome regulatory particle non-ATPase 13 (RPN13). This suppresses proteasome-catalyzed peptide splicing that reduces antigen cross-presentation to CD8^+^ T cells *in vitro* and in mice.^191^

The O-antigen of *Shigella* LPS may function as an immunological decoy in multiple ways that dampens the adaptive immune response. First, LPS is serotype specific. Second, the O-antigen is a carbohydrate that is a thymus independent type 1 antigen, which activates B cells without helper T-cell augmentation. This prevents class switching or a memory B-cell response to protect against re-infection.^192^ O-antigen specific antibodies are produced when LPS molecules are liberated from damaged bacteria at a low concentration, which can provide immunity to *Shigella* infection; however, they are short-lived and defeated with serotype conversion. The high concentration of O-antigen found on the surface of the bacteria leads to non-specific polyclonal B cell activation and the production of antibodies that do not confer protection. Thus, the acquisition of the SHI-O pathogenicity island may confer upon *Shigella* the ability to evade host immunity in more ways than one through LPS modification. To prevent an antibody response of any kind to its LPS, *Shigella* also induces apoptotic B-cell death, mediated by IpaD.^189^

## Persistence

*Shigella* typically causes a short-term infection; however, long-term carriage is possible.^193^ During long-term infection, *Shigella* acquires drug resistance and undergoes large chromosomal structural variations and rearrangements mediated by insertion sequences.^193^ Outbreaks of *Shigella sonnei* and *Shigella flexneri* with extensive multi-drug resistance including the ability to withstand fluoroquinolones and macrolides are on the rise, which curtails treatment options for severe infections.^194,195^

### Role of allelic variants in infection dynamics

OspZ reinforces the emerging theme in infectious disease of different alleles of the same gene fulfilling different roles in virulence and, in some cases, remarkably having opposed effects on the same process. A naturally occurring truncated OspZ allele found in some *S. flexneri* strains plays a role in intensifying the inflammatory response. In other strains, the full-length OspZ protein attenuates inflammation similar to its EPEC NleE homolog.^60^

### Campylobacter

*Campylobacter* enteritis is primarily caused by *Campylobacter jejuni* but also *Campylobacter coli* and occasionally by other species such as *Campylobacter lari, Campylobacter upsaliensis* and *Campylobacter fetus. Campylobacter jejuni* is a commensal bacterium in many animals that is especially common in poultry flocks that can cause diarrheal disease in humans when a potentially low dose of as little as 800 CFU is consumed with contaminated food or water. Diarrheal disease caused by this organism is a world-wide major public health issue. In 2017, *C. jejuni* surpassed *Salmonella* as the leading cause of food-borne bacterial diarrheal disease in the United States as it is in many European countries.^196^ *Campylobacter* activates and evades host immune responses in ways that directly benefit the pathogen and either directly or indirectly harm the host. Interestingly, numerous postinfectious disorders are associated with resolved campylobacteriosis including the development of Guillain-Barré syndrome, reactive arthritis, and irritable bowel syndrome. This may be attributable to the dysregulation or misdirection of the host immune response.^197^ This section emphasizes how *Campylobacter* overcomes innate barriers to infection and evades the adaptive immune system. For a more detailed discussion of *Campylobacter* pathogenesis and the host response, the reader is directed to additional reviews.^197–200^

## Animal models of disease

Small animal models of *C. jejuni* enteritis and bloody diarrhea have been somewhat difficult to establish as wild-type mice are colonized by the pathogen inefficiently and inconsistently. Ferret and chick models have been used, but they require genetic manipulation of the bacteria to mimic the enteritis seen in humans, limiting their utility to understanding aspects of disease beyond colonization.^201–204^ Infection of severe combined immunodeficient mice with a defined limited microflora or the introduction of microbes into antibiotic treated mice fed a zinc or protein deficient diet allows intestinal and systemic inflammation and has been used as models of human disease.^205,206^

## Overcoming colonization resistance

Following ingestion, *C. jejuni* colonizes the mucous layers of the epithelium of the lower intestinal tract. The intestinal microflora contributes metabolites that stimulate the expression of *C. jejuni* colonization factors and promote growth.^207^ Some *Campylobacter* strains enhance colonization by evading innate defenses with a type 6 contact dependent secretion system that delivers effector proteins into both prokaryotic and eukaryotic cells.^208^ These effectors mediate resistance to oxidative stress and provide a competitive advantage over the microflora and were also found to be important for *in vivo* survival.^208^

## Overcoming physical barriers

After penetrating the mucus and reaching the apical face of the epithelium, C. *jejuni* can pass either between or through individual intestinal epithelial cells to reach the basolateral side. Whether the bacteria transmigrate through the gastrointestinal epithelium exclusively paracellularly or transcellularly or both is unclear.^183^ *C. jejuni* secretes a serine protease, HtrA, which cleaves E-cadherin, occludin and claudin-8 to destabilize tight junctions, which enables the bacteria to transmigrate using a paracellular mechanism.^209–211^ Interestingly, HtrA proteins of other enteric pathogens including *Shigella, Helicobacter* and EPEC also cleave E-cadherin.^212^ Following transmigration, the bacteria adhere to the fibronectin-integrin complex that connects the epithelium with the underlying tissue with the adhesin CadF/FlpA and then invades the basal face of the epithelium.^209^

There is also evidence that *Campylobacter* invades the apical face of the epithelium and traffics within a *Campylobacter* containing vacuole to reach the basolateral side of the colonocytes and then exocytoses to the underlying tissue through a mechanism that is poorly understood. In one study, *C. jejuni* was found to invade epithelial cells and translocate through them following the sialylation of *C. jejuni* lipooligosaccharide structures, which generates human nerve ganglioside mimics.^213^ This may be of clinical relevance as ganglioside-like lipooligosaccharide-expressing isolates often cause severe gastroenteritis.^213^

A variety of studies provided conflicting results on the ability of *Campylobacter* to transmigrate through an epithelial monolayer with some reporting transcellular and other paracellular routes while others observed both.^214–221^ Most of these studies observed a time delayed drop in transepithelial electrical resistance.^214–220^ The most significant delay was observed in a three-dimensional *in vitro* cell culture assay that is likely more physiologically relevant than the more conventional two-dimensional ones.^221^ The differences among the various studies are likely attributable to different time points being used as well as different strains being employed. One study used 10 fresh *Campylobacter* isolates and found 10-fold differences among them in their ability to transmigrate through an epithelium.^215^ Some isolates favored transcellular penetration of the *in vitro* monolayer, while others primarily passed through it paracellularly while others used a combination.^215^

Following transit through the epithelium via paracellular and/or transcellular routes, several *Campylobacter* species, including *C. jejuni, C. coli, C. fetus*, and *C. upsaliensis*, can cause sepsis in both the immunocompromised and the otherwise healthy.^222^ In addition to the lamina propria, *Campylobacter* has been recovered from the mesenteric lymph nodes, blood and hepatosplenic tissue of infected animals.^223–227^ *C. jejuni* presumably benefits from colonizing the submucosa because it prevents their premature expulsion from the gastrointestinal tract by peristaltic forces and also may provide the bacteria with access to more nutrients. The pathogen can also contact basal receptors such as fibronectin and may be more protected from antibiotics as compared to the gut lumen. Also, by traversing the epithelium, *Campylobacter* can induce inflammatory diarrhea that enhances transmission to new hosts.

It is interesting to consider how and why *Campylobacter* sometimes reaches systemic sites and what the future may hold for the evolution of *Campylobacter* virulence. In one intriguing study, an unidentified extracellular *Campylobacter* factor(s) promoted the translocation of non-pathogenic bacteria not only through the gut epithelium but also to the blood, spleen and liver of mice.^220^ This may have implications for understanding and treating autoimmune disorders associated with *Campylobacter* infections. The passage of live non-pathogenic bacteria to systemic sites suggests a constitutive host directed antigen sampling pathway stimulated by a virulence factor as previously proposed for *Salmonella*.^37^

It is not clear why *Campylobacter* in some instances enters the bloodstream and colonizes systemic organs. In the case of *Salmonella*, it diverged from *Escherichia coli*, its nearest non-pathogenic relative to the acquisition of *Salmonella* pathogenicity island-1 and became a gastrointestinal pathogen.^228^ It is hypothesized to have become capable of systemic virulence when it later acquired *Salmonella* pathogenicity island-2 associated genes.^228^ Two to five percent of systemic infections with typhoidal serovars of *Salmonella* become asymptomatic within internal organs, with intermittent shedding, potentially for the lifetime of the host.^128,229^ Thus, the driving force in its ability to reach the bloodstream and the internal organs that filter the blood may have been to allow a longer-term association with the host. Perhaps while still mostly a short-term gastrointestinal pathogen, some strains of *Campylobacter* are following a similar evolutionary trajectory to that taken by some serovars of *Salmonella*.

## Activation and suppression of inflammation

*Campylobacter* exhibits numerous traits that facilitate colonization by directly subverting innate immune defenses that modulate inflammation. It possesses several phase-variable loci that modify cell surface structures, such as the capsular polysaccharides or lipooligosaccharides. Base deletion, insertion and single-nucleotide mutations lead to further inter- and intra-strain diversity of cell surface structures that allow subdivision into serotype. Collectively, this variation in enzymes and transferases changes resistance and immunogenicity and helps the bacteria evade the host’s immune system.^230^

*C. jejuni* eludes flagellum dependent TLR5 recognition and ensuant inflammation by carrying mutations in the eight amino acid epitope common to many bacterial pathogens’ flagellin. Interestingly, introducing this variant into *Salmonella* destabilizes the filament and eliminates motility. *Campylobacter* overcomes this by stabilizing the filament with new contacts not seen in other flagellar filaments.^231^

Intracellular *C. jejuni* can be detected with NOD1 and NOD2, which activate antibacterial defenses that are effective in limiting colonization by the microbe. Intriguingly, *C. jejuni* transitions from helical to coccoid peptidoglycan under times of stress, such as when they are within host cells that result in less NOD1 and NOD2-directed cell activation and inflammatory signaling.^232^ *Campylobacter* lipoproteins and LPS, however, are sensed by other Toll-like receptors, which activate NF-κB, resulting in the production and secretion of numerous pro-inflammatory cytokines including IL-8, which recruits neutrophils to the site of infection.^62^ The involvement of bactericidal neutrophils and other polymorphonuclear leukocytes can damage the intestinal epithelium and produce characteristic bloody diarrhea, which promotes bacterial dissemination to new hosts.

*Campylobacter* lacks numerous genes common to other enteric bacteria, notably including certain toxins and a canonical type III secretion system. The Cia proteins (*Campylobacter* invasion antigen) are introduced into the cytosol of epithelial cells with the flagellum export apparatus, which is ancestrally related to type III secretion systems.^233^ The secretion of these proteins is host cell contact dependent. Roles in promoting adhesion, invasion and intracellular survival have been attributed to them and thus they are essential for colonization.^234^

CiaC enhances invasion by mediating host cell cytoskeletal rearrangements that contribute to membrane ruffling necessary for internalization.^233^ *Campylobacter* remains confined within a *Campylobacter*-containing vesicle inside of the epithelium that it prevents from fusing with lysosomes.^62^ Once internalized into epithelial cells, CiaD activates the MAPKs to enhance the inflammatory response that includes additional IL-8 secretion from epithelial cells, which potently recruits neutrophils.^62^ At least one of the ∼18 secreted Cia proteins attenuates cytokine production rather than enhancing it *C. jejuni* in fact activates a PI3K/Akt-dependent pathway in human intestinal epithelial cells that is anti-inflammatory.^104^ By comparison with other enteropathogens, there is likely much more to be discovered in this area.

Some *C. jejuni* strains produce a genotoxin inside of host cells termed cytolethal distending toxin (CDT). CDT exhibits features of a type I deoxyribonuclease that is an important virulence factor. It arrests the cell cycle in G2/M transition phase. Cell division is inhibited but the cytoplasm continues to grow and distend, resulting in cells up to five times their normal size that remain viable for extended periods of time, before ultimately undergoing cell death.^235^ CDT was more recently proposed to be a unique and particularly potent virulence factor that acts as a tri-perditious toxin that impairs host defenses by disrupting epithelial barriers, suppressing acquired immunity and by promoting pro-inflammatory responses.^63,64^ Additional mechanisms may damage host DNA as some *C. jejuni* strains that lack CDT can still damage DNA and cause disease. A fascinating example is the recent discovery of *C. jejuni*-derived outer membrane vesicles that contain guide-free Cas9, which transcriptionally reprograms epithelial cells by activating DNA damage and NF-κB signaling and cell death pathways.^65,66^

### Persistence

Antimicrobial resistance represents a persistence strategy utilized by many enteric pathogens including *Campylobacter*. The prevalence of antimicrobial resistance in *Campylobacter* is increasing in humans and livestock. Like other bacteria, *Campylobacter* has developed various resistance mechanisms including ones that confer resistance to several classes of anti-microbial agents, which are of particular concern. One example of many is a mutant allele of *cfr* that methylates 23s rRNA in a manner that provides the bacteria with resistance to four different classes of anti-microbials.^236^ This gene is plasmid-borne and thus readily transferable through horizontal gene transfer. Other genetic factors of particular concern are the multiple drug resistance genomic islands of *Campylobacter* that are transferable between *Campylobacter* organisms via natural transformation and multi-drug efflux systems.^236^

Host nutrition plays an important role in determining if *Campylobacter* infection is acute or chronic. *Campylobacter* infection is more persistent and tends to be endemic in developing regions, whereas in contrast, in more developed areas, it more often presents as an acute, inflammatory illness.^197^ In the latter, the diet consists of a relatively higher proportion of processed foods that are mostly devoid of fiber. The dietary differences may result in people in less developed areas experiencing less intense inflammation because of a relative increase in colonic microbial fermentation due a fiber rich diet that results in greater short-chain fatty acid production which is anti-inflammatory.^237^ Additionally, postinfectious disorders associated with excessive inflammation occur more frequently in the developed world.^197^

## Role of allelic variants in infection dynamics

*Campylobacter* allelic variants that determine the degree of enteritis and the potential for extraintestinal dissemination have been identified with a variety of approaches.^238,239^ In one such study, amino acid substitutions within the *porA* gene were identified that are associated with extraintestinal disease. Lys189 is conserved among extraintestinal strains, and Asn189 is found in *porA* alleles associated within intestinal disease.^238^ This single amino acid change may alter the topology of the membrane domain in a way that allows the strains harboring this allele to evade recognition by the immune system. It may also facilitate ion transport to enhance bacterial metabolism.^238^ The discrepancies regarding the role of *porA* in causing disease may be related to the specific alleles that were present in the strains being studied.^238^

### Yersinia

In the genus *Yersinia*, three species are well-described human pathogens. *Yersinia enterocolitica* is ingested with contaminated food and can cause acute enteritis, enterocolitis, mesenteric lymphadenitis, and terminal ileitis and occasionally sepsis. It is the third most common foodborne zoonotic disease reported in European countries.^240^ *Yersinia pseudotuberculosis* is also inadvertently ingested with contaminated food and usually causes self-limiting mesenteric adenitis, ileitis and diarrhea but can also invade the bloodstream. Yersinia *pestis*, on the other hand, is a zoonosis transmitted to humans by fleas that causes the fatal systemic disease plague. *Yersinia* is primarily extracellular pathogens that mostly reside within the lymphoid tissue. Pathogenic species resist phagocytosis by professional phagocytes with a type III secretion system and suite of immunomodulatory type III effectors referred to as Yops encoded on a virulence plasmid. This section provides an overview of how *Yersinia* manipulates inflammation and a discussion of literature pertaining to the mechanisms underlying its persistence within hosts as well as its modes of extraintestinal dissemination. For a more detailed discussion of *Yersinia* pathogenesis and the immunomodulatory Yops, other reviews are recommended.^241–243^

### Animal models of disease

*Y. enterocolitica* and *Y. pseudotuberculosis* pathogenesis are typically studied in rodents, including BALB/c, C57Bl6 mouse strains and rabbits.^244^ Numerous mouse strains are highly susceptible to infection and the *ity* locus (Nramp) does not confer protection as it does in the case of *S*. Typhimurium and other pathogens.^245^ In murine models, the bacteria can cause gastroenteritis, adenolymphitis and septicemia with hepatosplenic tissue colonization. They can also cause a persistent infection and can be used to model *Yersinia*-associated reactive arthritis.^246,247^

### Overcoming colonization resistance

*Yersinia* binds to the mucus with the adhesin YadA and thins and degrades it with enzymes.^248^ *Yersinia* appears to scavenge iron better than commensal bacteria, giving it an advantage, although it appears to be sensitive to colicins, which are bactericidal proteins that are continuously released by the resident microflora.^248^ *Yersinia enterocolitica* has genes for tetrathionate reduction and may use it as an electron acceptor in the inflamed intestine, similar to *Salmonella*.^20^ *Yersinia* can bind to the epithelium with YadA and invasin.^249,250^ Overall, research in overcoming colonization resistance by *Yersinia* is lacking compared to other enteropathogens and in need of additional studies.

### Overcoming physical barriers

After penetrating the mucus, *Y. pseudotuberculosis* and *Y. enterocolitica* bind the ß1 integrins of M cells with the outer membrane protein invasin, which triggers an internalization event.^251^ *Yersinia* is transported within a vacuole in the cytosol of epithelial cells to the basal side of the epithelium from which it exits and is conventionally believed to resist phagocytosis by the underlying professional phagocytes. The infection can proceed to the regional lymph nodes and is believed in some cases to drain through the thoracic duct into the bloodstream. However, there is evidence of *Yersinia* being transported to the mesenteric lymph nodes by CD103^+^ dendritic cells in a CCR7-dependent manner.^252^

While enteropathogenic *Yersinia* reside within lymphatic tissue, the sequential ordered spread of these pathogens through the lymphatic system to the blood has been challenged in a pair of studies.^33,252^ In one, the authors observed that *Y. pseudotuberculosis* disseminated to the blood and subsequently internal organs following replication in the intestine of mice independently of the lymphatic system.^33^ The authors reported that *Y. pseudotuberculosis* colonized the hepatosplenic tissues of mice genetically devoid of Peyer’s patches identically to congenic control mice, as was also observed with *Salmonella*, indicating the availability of alternative routes of extraintestinal dissemination for enteropathogens.^13,15,17,33,39,40^

In another more recent study, *Y. enterocolitica* was likely transported by reverse transmigrating CX_3_CR1^+^ phagocytes associated with the lamina propria to the spleen independently of Peyer’s patches. The authors observed that the association of the bacteria with these host cells was dependent on invasin but independent of YadA.^252^ This study used a 1-hour time point; however, in the former one by Barnes et *al*., the rapid colonization of hepatosplenic tissue following oral inoculation was resolved. A temporally distinct delayed translocation (out to 1 week) that did not involve the lymphatic system resulted in successful colonization of the spleen and liver. Whether or not transport from the lamina propria to the spleen and liver by CX_3_CR1^+^ phagocytes involves reverse transmigration needs to be determined and a time course is needed.

Barnes *et. al*. addressed the possibility that blocking one pathway of extraintestinal dissemination may merely result in more bacteria disseminating through other ones. In an experiment in which none of the pathways were blocked, clonal analysis of uniquely tagged *Y. pseudotuberculosis* strains revealed that bacteria that successfully colonized the hepatosplenic tissue were not derived from the Peyer’s patches or the mesenteric lymph nodes. Rather, they translocated directly from a replicating pool in the lumen of the intestine out to 1 week post-oral infection.^33^ As is the case with *Salmonella*, more study is needed to clarify the routes of extraintestinal dissemination exploited by *Yersinia*.

### Activation and suppression of inflammation

*Yersinia* actively suppresses microbial internalization by microbicidal phagocytes with YopE and YopT. YopE stimulates the hydrolysis of GTP associated with the Rho GTPases Rac1, RhoA, and Cdc42, to render them in their inactive GDP-bound form.^67,68^ YopT, on the other hand, is a cysteine protease that disrupts RhoA activity by disassociating RhoA from the plasma membrane by cleaving the prenyl group.^70–72^ By interfering with RhoA GTPase activity, YopE and YopT activate Pyrin.^69^ Pyrin is an inflammasome NLR highly expressed by phagocytes that is activated when RhoA GTPase activity is modulated, which is a shared tool through which extracellular pathogens prevent their phagocytosis.^253^

The sensitivity of *Yersinia* to pyrin-mediated inflammation is evidenced by *Yersinia* secreting YopM into phagocytes, which blocks pyrin activation and is essential for virulence.^106^ YopM is a leucine-rich repeat effector protein that is harbored by all strains of *Yersinia* that are pathogenic to humans. Its loss reduces colonization, increases host inflammation and often allows the host to survive.^106^ Macrophages infected with a *Yersinia yopM* deletion strain have an increased level of activation, IL-1β secretion, and host cell death compared to those infected with wild-type strains.^106,107^ YopM inhibits caspase-1 activation by subverting host kinases that are negative regulators of pyrin, which down regulates the pro-inflammatory cytokines IL-1β/IL-18.^105^ This allows *Yersinia* Yops to block phagocytosis with little consequence. Macrophages infected with *yopM* deletion mutants initiate a strong pyrin inflammatory response, demonstrating the importance of YopM to infection and mice infected with them are more likely to survive than those infected with wild-type *Yersinia*.^106^ On the other hand, mice that lack pyrin are susceptible to Δ*yopM Yersinia*.^106^ Thus, pyrin inflammasome activation limits *Yersinia* persistence, but YopM counters it.

YopK/YopQ is a type III effector that partially blocks inflammasome activation indirectly by regulating the translocation of other Yops into host cells.^108^ YopK prevents hyper translocation of the T3SS pore-forming proteins YopB and YopD, but this does not prevent entry of low basal levels of these proteins, which is associated with inflammasome activation.^108,109^ Indeed, the magnitude of inflammasome activation appears to be a function of the relative amount of YopB and YopD introduced into the host cell, as increasing levels of YopD in the host cytosol correlate with larger caspase-1 oligomers.^109^ YopK affects the size of pores introduced into the host cell membrane. YopK interacts with both YopB and the host protein RACK1 but it is not known how YopK regulates translocon pore structure.^107^ Both canonical and noncanonical inflammasomes are activated when cells are infected with a *yopK* mutant.^107^ Enteropathogenic *Yersinia yopK* mutants can colonize the Peyer patches, but mice infected with these mutants mount an unusually early inflammatory response that prevents microbial dissemination to the spleen and liver.^254^ Studies of YopK establish the importance of bacterial virulence factor regulation in dampening type III secretion system-induced innate immune signaling. However, while all organisms that harbor a type III secretion system have YopB and YopD orthologs, no YopK orthologs are known to exist outside *Yersinia*.

### Modulation of host cell death

The type and mechanisms of host cell death induced by *Yersinia* infection are unclear with reports of necrosis, pyroptosis and apoptosis. YopJ/YopP inactivates the MAPKs with its acetyltransferase activity, which effectively blocks NF-κB and MAPK signaling. Importantly, when NF-κB/MAPK signaling is uninhibited, it allows the expression of proinflammatory and pro-survival genes, enforcing cell survival during infection. In fact, YopJ is indispensable for *Yersinia*-induced rapid killing of macrophages.^255,256^ The targeting of immune cells by the type III secretion system might be expected to render these cells ineffectual in responding to *Yersinia* infection. However, this is not the case; instead, YopJ interdiction of MAPK and NF-κB signaling trips a host pattern-of-pathogenesis alarm that triggers a unique cell death response in infected macrophages. This type of cell death was proposed by one group to involve PANoptosis.^257,258^ PANoptosis is an inflammatory programmed cell death pathway that shares features of necrosis, pyroptosis and apoptosis and features crosstalk between the respective signaling complexes.^257,258^ More work will be required to clarify the type of cell death induced by *Yersinia* and to delineate the underlying mechanism.

### Role of allelic variation in infection dynamics

An emerging theme in infectious disease is for pathogens to possess more than one allele of the same gene that in some instances differ by only a single nucleotide polymorphism that changes infection dynamics. Despite the conservation of *yopJ* in the human-pathogenic *Yersinia* lineage, the role of YopJ-induced cell death in virulence is not clear with some studies reporting that it contributes to virulence with others finding that it attenuates it. A recent report found that a conservative substitution at codon 177 that encodes a leucine in *Y. enterocolitica*, codes for a phenylalanine in nearly all of the more pathogenic *Y. pseudotuberculosis* and *Y. pestis* strains. The change, despite being conservative, renders YopJ less cytotoxic to macrophages, yet more virulent.^259^ *Yersinia pseudotuberculosis* engineered to express YopJ Leu177 in a mildly attenuated *ksgA* mutant background was completely avirulent in mice.^259^ YopJ is one of the numerous genes in enteropathogens for which different naturally occurring alleles have recently been discovered that dictate how the pathogens harboring them interact with the immune system.

### Persistent infections

Low levels of *Yersinia* species are frequently found in the human ileum that are not often associated with disease. Perhaps surprisingly, 7.6% of people analyzed by a highly sensitive method were asymptomatic *Yersinia* carriers.^260^ This was a small study and a larger scale one to assess what percentage of the population asymptomatically carries various gut pathogens would be interesting. Such infections are a public health concern because they may lead to autoimmune disorders and can serve as reservoirs in which the pathogens can evolve and disseminate to more susceptible hosts.^261^ *Y. pseudotuberculosis* can cause persistent infection in mice, where it remains associated with the lymphoid follicles of the cecum.^247^ In this model, infecting with a low dose of bacteria leads to asymptomatic infection in 20–30% of mice with carriage for as long as 115 days. Even if no signs of disease are present, *Y. pseudotuberculosis* persistence is associated with an immune response in which the bacterial foci are surrounded by polymorphonuclear neutrophils and this is accompanied by prolonged fecal shedding.^247^

*Yersinia, Salmonella*, and *Campylobacter* have all been reported to infect and affect the ileocecal area in humans, suggesting that the cecum is a beneficial niche for bacterial persistence.^247^ The mechanisms enabling *Y. pseudotuberculosis* to persist in cecal tissue in the presence of immune cells for a prolonged time are largely unknown. YopH and YopE contribute to *Y. pseudotuberculosis* persistence in the cecum, likely by enabling initial colonization in the presence of phagocytic cells. Later, the bacteria become persistent with a novel expression profile, suggesting substantial transcriptional reprogramming. Genes on the virulence plasmid are highly up-regulated initially that in the chronic stage are down-regulated, perhaps to allow persistence in the face of an ongoing immune response. In association with the latter stage, genes involved in anaerobic growth, motility, protection against acidic and oxidative stress as well as genes indicating envelope perturbation are upregulated, suggesting adaptation to the harsh environment in cecal tissue.^262^ Along with considerable transcriptional reprogramming, the copy number of the *Y. pseudotuberculosis* virulence plasmid changes during infection. An early increase in copy number is essential for virulence. A later attenuation in gene dosage may contribute to persistence by reducing the chance that the immune system responds to the Yops and is able to ultimately clear the infection.^263,264^

Persistent *Yersinia* infection mimics and some have proposed plays a role in Crohn’s disease. Both yersiniosis and Crohn’s disease are characterized by persistent reactivity against the gut microbiota.^265^ Like yersiniosis, the inflammation characteristic of Crohn’s disease is initially focused around the follicle-associated epithelium and then may progress into deeper, more diffuse ulcerations. Lymphatic vessels have been proposed to play key roles in both the dissemination of yersiniosis and Crohn’s disease.^266^

## Vibrio

*Vibrio cholerae* is one of the most consequential pathogens in human history. It is responsible for many pandemics of severe diarrheal disease that has produced case fatality rates of 50% or more, with death often occurring within hours of the onset of symptoms.^267^ *Vibrio*s are natural pathogens of both human and aquatic animals that are commonly found in both freshwater and marine environments.^268,269^ Of the greater than 100 species, about 12 are pathogenic to humans. The most common *Vibrio* species that cause disease in humans are *Vibrio cholerae, Vibrio parahaemolyticus, Vibrio vulnificus* and *Vibrio alginolyticus. V. cholerae* is typically contracted through the consumption of contaminated seafood or water and is the etiological agent of the severe diarrheal disease known simply as cholera. Other *Vibrio* species, such as *V. parahaemolyticus* and *V. vulnificus*, are responsible for a group of infections termed *Vibriosis* that have different clinical manifestations. The specific disease caused varies by species, route of infection and host susceptibility and includes nothing more than gastroenteritis but they can also cause primary septicemia. Additionally, they can colonize skin wounds, which may lead to secondary septicemia.

Cholera remains an important public health challenge, despite the development of effective vaccines, with an estimated 5 million cases globally on an annual basis with approximately 100,000 fatalities and has been endemic in Asia for centuries, which continues to this day.^270^ The public health threat may be heightened in coming years by increasing temperatures associated with climate change as *Vibrio* thrives in warm water.^269^ The numerous *Vibrio* species are diverse but share several notable features including, among others, genomes that are composed of two circular chromosomes that have been extensively shaped by horizontal evolution.

The *V. cholerae* species is subdivided into over 200 serogroups with two prominent ones, O1 and O139, responsible for epidemic cholera. The O1 serogroup is composed of two biotypes, classical and El Tor. The classical and El Tor biotypes differ in many phenotypic traits. The classical biotype caused the first six cholera pandemics, while the El Tor biotype is responsible for the ongoing seventh pandemic.^268^ In recent years, there has been an increase in the number of infections with other serotypes of *V. cholerae* with most causing mild gastroenteritis, ear and wound infections or bacteremia in the immunocompromised; however, extraintestinal infections including fatal bacteremia in otherwise healthy patients do occur.^271^ This section discusses how *Vibrios* overcome colonization resistance and their intriguing interactions with the resident gut microflora. It also describes the effects of *Vibrios* on the inflammatory response. For comprehensive reviews of *Vibrio* pathogenesis, additional reading is recommended.^268,272–274^

### Animal models of disease

The most frequently used model of *V. cholerae* pathogenesis is the infant mouse model. Typically, following oral inoculation, within 16 hours, a diarrheal illness manifests, similar to human infection that requires cholera toxin.^275^ The scarcity of biological materials present in this model for transcriptomic studies and genome-wide functional screens often results in the utilization of an infant rabbit model.^275^ *Drosophila melanogaster, Caenorhabditis elegans* and *Danio rerio* models have also been developed.^276–278^

### Overcoming colonization resistance

*V. cholerae* is the best understood of the *Vibrio* species that are pathogenic to humans. It is a non-invasive pathogen whose preferred niche is the mucosal surface of the intestine. The host microbiome has long been suspected to play a role in susceptibility to *V. cholerae* infection as the household contacts of cholera patients are known to be at elevated risk of infection. Moreover, in animal models of infection, the normal microbiota must be disrupted with antibiotics to render them susceptible to *V. cholerae*.

*V. cholerae* employs multiple strategies for overcoming the formidable innate defense of endogenous microflora that results in a multi-log reduction of resident microbes during infection. First, the pathogen has a faster growth rate than members of the commensal microflora giving it a competitive advantage. Secondly, *V. cholera* deploys a type VI secretion system in response to environmental cues found in the intestine that kills adjacent bacteria but shields the pathogen from its own bactericidal effectors.^279^ A third feature of *V. cholerae* pathogenesis that dramatically reduces the density of members of the resident microflora is the profuse watery diarrhea that is induced and the destruction of the mucus layer. A fourth feature of *V. cholerae* infection that benefits the pathogen at the expense of colonic microbes is the activation of the innate immune system that is ineffective in responding to the initial infection to which the microflora is sensitive. This involves NF-κB and MAPK activation as well as Toll-like receptor signaling and the release of bactericidal proteins. The ensuant immune response includes the generation of reactive oxygen species such as dual oxidase 2 and nitric oxide synthase, which *V. cholerae* counters with specific inducible resistance mechanisms that the microflora lacks.^280^

The characteristic profuse, watery diarrhea associated with *V. Cholerae* infection is primarily attributable to its primary virulence factor, cholera toxin. The cholera toxin B subunit binds to a carbohydrate on the surface of host cells that triggers the internalization of the A subunit, which mediates cAMP-mediated activation of anion secretion and fluid loss. *V. cholerae* secretes various other enzymes and toxins that promote its persistence within the gastrointestinal tract. This includes Hap, a secreted Zn^2+^-dependent metalloproteinase that has mucinolytic activity that promotes mucin penetration and the spreading of infection along the gastrointestinal tract.^281^ The toxin zonula occludens disrupts epithelial integrity by interacting with tight junction proteins.^282^

Cell curvature is another important feature of *V. cholera* pathogenesis that aids in persistence. A periskeletal element, CvrA, inserts more peptidoglycan in the outer face of the bacteria than the inner face. This asymmetrical patterning of peptidoglycan produces the curvature, which is characteristic of all *V. cholera* isolates studied to date and enhances host colonization.^283^

*Vibrio* tightly regulates and coordinates the expression of its arsenal of virulence factors with the transcriptional regulator ToxT. ToxT is regulated by environmental signals and up-regulates cholera toxin, the toxin co-regulated pilus (Tcp) and a type IV pilus, which are essential for persistence. The TCP facilitates intestinal colonization by promoting microcolony formation by mediating interaction between adjacent bacteria through direct pilus to pilus contacts, tethering them together as well as to enterocytes and is essential.^284^

Several recent studies utilized metagenomics and gut microbiome transplantation into gnotobiotic mice to reveal intriguing aspects of the complex interactions that occur between *V. cholerae* and the host gut microbiome. Some species that can be found in the commensal microflora of humans promote the virulence of this pathogen while others suppress it. In two such studies, interpersonal differences in the human gut microbiota were shown to predict susceptibility to *V. cholerae* infection, with depleted levels of Bacteroidetes microbes rendering one vulnerable and species within the genera Prevotella and Bifidobacterium being protective.^285,286^ *Blautia obeumcan*, a commensal species in the gastrointestinal tract of healthy humans, was shown in another to suppress *V. cholerae* colonization by degrading taurocholate, a host-produced virulence-inducing molecule.^287^ *Ruminococcus obeum* was demonstrated to restrict the expansion of *V. cholerae* in the gut of mice by producing a signaling molecule that suppresses several of these pathogen’s colonization factors that are regulated by quorum sensing.^288^ Intriguingly, *Paracoccus aminovoran*s forms a multi-species biofilm with *V. cholerae* that facilitates its colonization of the gut.^289^ For more information on how *Vibrio* interacts with the gut resident microflora, readers are referred to other reviews.^267,290^

### Activation and suppression of inflammation

*V. cholerae* directly engages the innate immune defenses of the colonic epithelium, both activating it and suppressing it to its advantage. The pathogen disrupts its integrity with cholera toxin and further activates the inflammatory response to its own advantage with the pore-forming toxin cytolysin, encoded by the *hlyA* gene. The cytolysin is a ß-barrel pore forming toxin that induces mitochondria-dependent apoptosis.^73^ It results in inflammation in part by stimulating the NF-κB and MAPK pathways.^74^ *Vibrio cholerae* secretes Hap, a Zn^2+^-dependent protease that has mucinolytic activity to neutralize the intestinal mucus layer. It produces another toxin, zonula occludens (ZOT), to disrupt epithelial integrity by manipulating the tight junction proteins occludin and zonula occludens 1 protein (ZO1).^281,291^ *V. cholerae* blunts the inflammatory response of the epithelium provoked by cytolysin with virulence factors that increase levels of microRNA miR-146a, which has immunomodulatory effects, that are delivered to host cells with outer membrane vesicles.^75^ By releasing these outer membrane vesicles with cargo that both activate and dampen the inflammatory response of the epithelium, the pathogen can evade innate immune defenses allowing severe disease and persistence. An intriguing observation was that *V*. Cholera appears to combat neutrophil extracellular traps with DNases.^110^

### Non-O1/non-O139 serogroup strains

Although they have similar clinical outcomes, pathogenic non-O1/non-O139 serogroup strains achieve them with different virulence factors than epidemic associated O1 and O139 serogroup ones. The former possesses a more diverse collection of virulence factors than the latter, which are mostly clonal.^292^ Most clinical isolates belonging to non-O1/non-O139 strains lack cholera toxin and the toxin co-regulated pilus but achieve the same effect through less characterized mechanisms. Some of them encode a type III secretion system that helps them colonize the epithelium, disrupt its homeostasis and engage innate immune signaling pathways. The type III secretion system of these strains exerts cytotoxic effects on host cells via osmotic lysis following cortical membrane reorganization and disassembly of epithelial junctions, rather than by apoptotic or necrotic mechanisms.^292^ It is most similar in sequence with the chromosomally encoded *Yersinia* Ysc type III secretion system and the two systems from these enteric pathogens show a fair degree of synteny. A subset of *V. cholerae* non-O1/non-O139 serogroup strains which do not cause epidemic disease but do provoke diarrhea encode a second type III secretion system and lack the canonical virulence factors.^273^ Many *V. parahaemolyticus* strains express both type III secretion systems, one from each chromosome.^293^ Animal models of infection reveal that strains that encode both systems use the second one to promote colonization and disease.^273,294^ A second type III secretion system can also be harbored by *V. mimicus* and *V. anguillarum* that are pathogenic to humans.^295^

### Persistence

*V. cholera* usually produces a short-term infection that is typically treated with rehydration therapy; however, it is an enteropathogen notorious for multi-drug resistance, which is problematic in severe or prolonged cases of illness. A fascinating recent discovery was that CvrA mRNA is regulated by a sRNA that is in turn induced by cell-wall targeting antibiotics so that cell shape is optimized for resistance.^296^ Most drug resistance, however, is due to the genome plasticity of this pathogen. *Vibrio* frequently acquires extrachromosomal mobile genetic elements from not just closely but also distantly related species of bacteria that often allow it to resist antimicrobial agents.^297^ In fact, over time, *V. cholera* has acquired mobile genetic elements that confer resistance to all commonly used antibiotics.^297^

## Discussion

While considerable progress has been made in understanding infectious disease, many open questions and challenges remain. For example, we need to determine how to modulate the endogenous microflora to manipulate the complex interactions of enteropathogens with it to suppress infection. We also need to enhance our understanding of the spatiotemporal regulatory inputs that are integrated by pathogens to strike the delicate balance between the induction and suppression of inflammation by enteropathogens to optimize infectiousness and persistence. An exciting emerging trend is the differences being discovered among allelic variants of virulence genes that dictate how the enteropathogens harboring them interact with the immune system. We will need to determine how widespread this is and much follow-up on metagenomic and bioinformatic studies will be required to fully understand the allelic differences observed. These experiments may provide us with unique insight into the molecular mechanisms underlying the subversion of the immune system by pathogens and new tools for fighting them. Perhaps predictive models for what genetic changes are likely to cause outbreaks can be developed.

There are dozens of enteropathogenic secreted effectors with anti-inflammatory properties in addition to SpvC, many of which are required for disseminated infections.^87,125^ The requirement of many of these genes for lethal disease has historically been attributed to them blunting the innate immune system primarily by subverting killing mechanisms; however, enigmatically, many of them are dispensable for both intracellular survival and the inhibition of host cell death.^84,87,125^ A potentially important, although often overlooked, component of a pro-inflammatory response is the production and release of cytokines that potently inhibit the movement of infected cells. The field will need to determine which effectors with anti-inflammatory properties are required for virulence because in addition to or perhaps in some cases instead of attenuating more traditional host anti-microbial mechanisms, they manipulate the ability of infected cells to control their movement. For example, macrophage migration inhibitory factor, the release of which is an integral component of an inflammatory response, was initially described as a soluble factor that potently inhibits the migration of phagocytes.^298^

Chronic infections and the long-term sequelae associated with them are on the rise and just beginning to be understood. Their incidence is becoming more and more of a public health issue with the alarming increase in multi-drug resistance. Outbreaks of acute disease caused by multi-drug resistant bacterial enteropathogens have become so commonplace that some infections with them are essentially untreatable. A systematic network analysis of *Salmonella typhimurium* metabolism in the murine model of typhoid fever determined that nearly all *S. typhimurium* enzymes are non-essential, due to extensive metabolic redundancies.^299^ Not surprisingly, most microbial targets considered druggable are already interdicted with existing drugs.^299^ Thus, a final remaining challenge is the need to develop new drugs for treating infections caused by enteropathogens, preferably that the bacteria will be unable to quickly evolve ways to overcome.
